# EAPhy: A Flexible Tool for High-throughput Quality Filtering of Exon-alignments and Data Processing for Phylogenetic Methods

**DOI:** 10.1371/currents.tol.75134257bd389c04bc1d26d42aa9089f

**Published:** 2015-08-05

**Authors:** Mozes P K Blom

**Affiliations:** Department of Evolution, Ecology and Genetics, Australian National University, Acton, ACT, Australia

## Abstract

Recently developed molecular methods enable geneticists to target and sequence thousands of orthologous loci and infer evolutionary relationships across the tree of life. Large numbers of genetic markers benefit species tree inference but visual inspection of alignment quality, as traditionally conducted, is challenging with thousands of loci. Furthermore, due to the impracticality of repeated visual inspection with alternative filtering criteria, the potential consequences of using datasets with different degrees of missing data remain nominally explored in most empirical phylogenomic studies. In this short communication, I describe a flexible high-throughput pipeline designed to assess alignment quality and filter exonic sequence data for subsequent inference. The stringency criteria for alignment quality and missing data can be adapted based on the expected level of sequence divergence. Each alignment is automatically evaluated based on the stringency criteria specified, significantly reducing the number of alignments that require visual inspection. By developing a rapid method for alignment filtering and quality assessment, the consistency of phylogenetic estimation based on exonic sequence alignments can be further explored across distinct inference methods, while accounting for different degrees of missing data.

## Introduction

High-Throughput Sequencing (HTS) has revolutionised the field of phylogenetics by enabling researchers to question the evolutionary relationships between taxa with large-scale multi-locus datasets 1 ^,^ 2. The development of these methods has been driven by a realisation that the inclusion of many genetic markers helps to account for stochastic coalescent histories of individual genes 3 ^,^ 4 ^,^ 5 ^,^ 6. Species tree inference methods use the multispecies coalescent model to estimate potential gene tree – species tree discordance and large numbers of unlinked loci represent a greater sample of the gene tree distribution underlying the true species tree 6. However, while phylogenetic estimation might improve by sequencing many loci 4 ^,^ 5 ^,^ 6 ^,^ 7, the requirement for high-quality sequence alignments remains unchanged and is fundamental for the correct inference of phylogenetic hypotheses. Existing alignment methods can be extrapolated for use with large-scale multi-locus datasets, but visual inspection of each alignment, the traditional approach for assessing alignment quality, is challenging with thousands of sequenced loci 8. As a consequence of the impracticality of visual inspection, the impact of missing data in large phylogenomic datasets is often nominally explored and the potential consequences of distinct alignment filtering criteria remain unknown. Nonetheless, contradicting opinions coexist 9 ^,^ 10 ^,^ 11 regarding the effect of missing data on phylogenetic inference and it is therefore advisable to quantify the sensitivity of empirical phylogenetic hypotheses to data filtering choices. Thus we need workflows that automate (as far as possible) the assessment of alignment quality and the consequences (in terms of missing data) of making different choices about filtering criteria. Ideally, such a workflow would facilitate the conversion of individual contiguous sequences (‘contigs’) into quality-filtered alignments, and help to minimise the demand for visual inspection.

The need for a high-throughput alignment filtering system emerged with the recent advance in molecular methods to target and sequence large numbers of orthologous loci. Since whole-genome sequencing is still too costly for most research labs that focus on non-model organisms, genome reduction protocols have been developed that isolate large numbers of orthologous loci across the genome of closely related and deeply divergent taxa 1 ^,^ 8. There are two increasingly popular genome reduction methods that specifically focus on exonic sequence regions and can generate genetic markers suitable for phylogenomic inference. Transcriptome sequencing is a cost-effective method that does not require the a-priori availability of genomic resources. RNA is extracted from the same tissue in different target species and with the expected expression of similar genes, orthologous loci are isolated and sequenced for phylogenetic comparison. An alternative method, exon-capture 12, is a target enrichment approach 13 ^,^ 14 ^,^ 15 ^,^ 16 that benefits from an increasing number of readily available genomic resources and enables the design of study-specific capture systems. The use of exonic sequence regions for phylogenetic inference, generated by transcriptome sequencing or exon-capture, is promising and has been successfully demonstrated at different levels of divergence across the tree of life 17 ^,^ 18 ^,^ 19 ^,^ 20. The tremendous increase in the scale of available exonic loci benefit inference methods, but also requires a significant investment in the development of bioinformatic resources to process such data.

Whereas several excellent bioinformatic pipelines have been constructed for processing raw sequence data and conducting sequence assembly 13 ^,^ 14 ^,^ 15 ^,^ 16 ^,^ 19, a bioinformatic scheme is needed for subsequent alignment, alignment quality assessment and alignment filtering. Most published studies still conduct visual inspection of alignment quality and account for missing data by dividing datasets into a limited number of categories manually (i.e. 14 ^,^ 18)or automated (i.e. 21). Recently, Misof et al.^19^ developed a method to assess alignment quality in an extensive study that used transcriptomes to infer the phylogeny of insects. They identified potentially erroneous alignments by calculating the BLOSUM62 distance between each amino acid sequence and the best reciprocal hit of a reference taxon. A distance calculation based on a BLOSUM matrix was warranted, due to a significant level of protein divergence between most taxa. The BLOSUM alignment score matrix values the alignment of each amino acid pair differently, representing the likelihood of amino acid substitutions, but lacks resolution when the expected level of protein divergence between two sequences is limited. However, although this has not been tested prior, it can be expected that at shallower levels of divergence subtle misalignments might actually have more significant consequences for phylogenetic estimation than when inferring relationships between distant taxa, stressing the need to identify such misalignments. When assessing alignments with limited levels of sequence divergence, the exact number of clustered amino acid changes is more likely a better indicator of alignment quality than the overall BLOSUM62 distance score. In this short communication, I describe a flexible high-throughput pipeline for quality assessment of exonic sequence alignments and subsequent filtering of missing data. The pipeline is specifically designed to be flexible and process both population and phylogenetic level data, but the method developed by Misof et al.^19^ will likely be more effective at deep phylogenetic scales.

EAPhy, exon alignment for phylogenetics, was developed to process exonic sequence data for phylogenetic inference, but is valuable for any type of analysis that requires high-quality filtered alignments (i.e. population genomics or molecular evolution). In this manuscript I will focus on its application for phylogenetic inference. The first objective of the pipeline is to quantify alignment quality and highlight just those loci that require visual inspection. By translating exonic nucleotide alignments into amino acid alignments, EAPhy infers the relative quality of sequences and alignments by assuming that most mutations within exons are silent. In addition, the identification of regions that harbor an excessive cluster of amino acid replacements distinct from a summary reference sequence, is used as a proxy for alignment quality. Simultaneously, insertions and deletions that result in frame shifts and the introduction of multiple stop-codons are unlikely to represent true biological events and such alignments should be addressed. The pipeline can be adapted based on the expected level of divergence between taxa by adjusting the stringency of filtering criteria. The second objective is to provide a user-friendly method to account for missing data. By enabling filtering criteria for missing data to vary, the consistency of phylogenetic estimation can be quantified across different levels of missing data. Lastly, EAPhy was designed to generate alignments of different sorts (haplotype, diplotype and SNP based), in the formats required for most commonly used inference software and facilitate the further exploration of distinct analysis methods. With the development of a high-throughput method for alignment filtering and processing, the overarching aim of this pipeline is to reduce the bioinformatic burden of data analysis involving exonic sequence alignments and ultimately promote further research into the (in)congruences between inference methods, while accounting for different degrees of missing data.

## Overview of Methods

EAPhy consists of a collection of scripts that takes as input a set of unaligned sequences for an arbitrary number of species and loci. It will generate multiple sequence alignments using existing aligning software and subsequently filters these alignments for a number of user-specified criteria. The final output consists of quality filtered multiple sequence alignments, allowing different degrees of missing data as preferred, and a list of alignments that still require visual inspection. The output files are automatically exported in the input format of commonly used phylogenetic inference programs. The complete package is freely available at https://github.com/MozesBlom/EAPhy.

EAPhy can be run on most individual computers (i.e. does not require a cluster set-up) and an individual run for a modest dataset can be completed within hours. The pipeline has been used with exon-capture datasets involving tens to hundreds of individuals and thousands of loci, and finished within six hours on a Macintosh desktop computer with a 3.1 GHz Intel i7 processor (2012) and 16 GB of RAM. It is important for the user to adopt filtering criteria suitable for the dataset (level of divergence and data quality) analyzed, but if filtering criteria have been carefully reviewed EAPhy should be able to handle larger datasets than currently tested. For EAPhy to function appropriately, I advise to run the pipeline initially with a small subset of the data and replicate the analysis with alternative filtering criteria. If filtering and flagging of alignments works well, then the analysis can be extrapolated for usage with the complete dataset. The importance of specifying appropriate filtering criteria should not be underestimated, since misspecification of filtering criteria will result in a significantly reduced dataset or alternatively a dataset that equals the input data, regardless of potential low-quality alignments.

EAPhy is not designed to identify individual sequencing errors that are often associated with HTS datasets, but will identify sequence regions with excessive non-synonymous substitutions (potential ‘low-coverage’ sequences) if these have not been filtered out beforehand and appear anomalous in the resulting alignments. Several excellent pipelines have been developed to filter raw sequence data and generate assemblies, and the starting point of this pipeline requires assembled individual contigs that start in first codon frame, for each presumed orthologous locus. A complete overview of the pipeline is outlined in Figure 1 and a general description of the most important components is provided here.

***Specification of configuration script***

At the onset of each EAPhy run, the system path to an align program executable and all filtering criteria for downstream analysis are specified in a single configuration script. Muscle 22 is the default aligner used by EAPhy, but should be installed by the user independently from downloading EAPhy. Alternative alignment software can be used but requires modifications of several scripts. The EAPhy pipeline is designed as a set of modules that can be executed independently or in consecutive order as a complete analysis (Fig. 1). This provides a straightforward system to reiterate specific components of the pipeline, with alternative filtering criteria for alignment quality or missing data. A complete description of all filtering parameters can be found in the manual and is part of the EAPhy package that can be downloaded from GitHub.

**A schematic overview of the EAPhy workflow:**
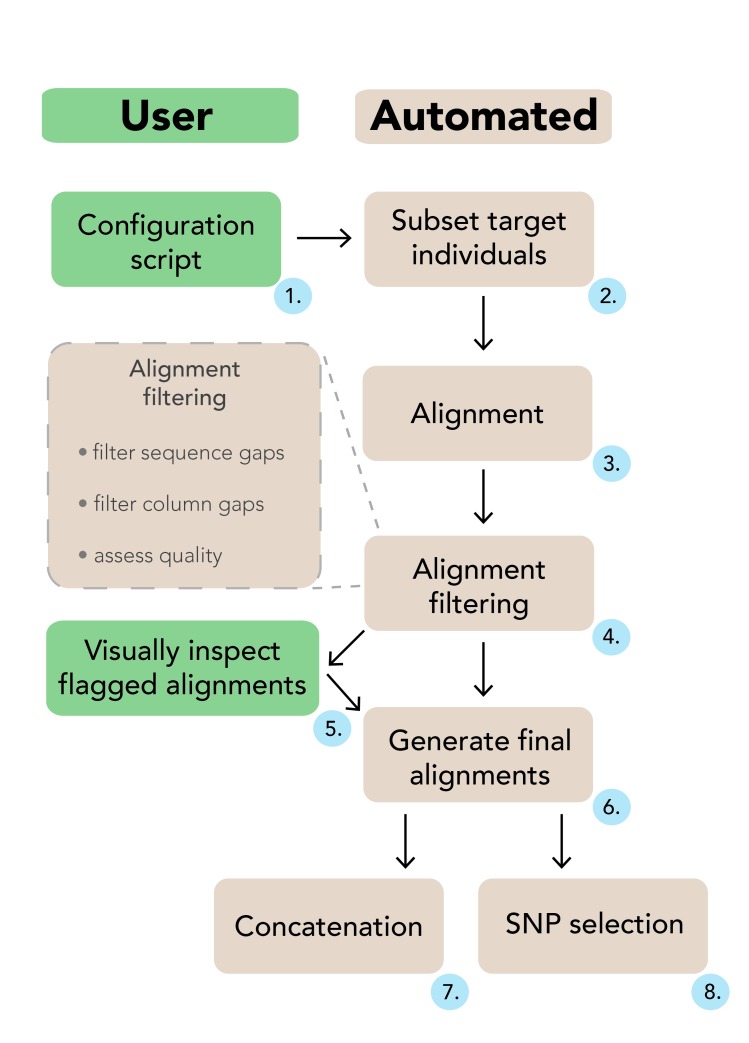
The user specifies filtering instructions in a single configuration script (1). EAPhy will first subset the target individuals from each locus and create new contig files (2). With an existing aligner, new alignments are created for each locus (3) and are subsequently processed and checked (4). Alignments are highlighted that do not fulfill the filtering criteria and can be visually inspected. If deemed appropriate for inclusion, manually checked alignments can be added to the filtered alignment list (5). Alternatively, EAPhy can automatically continue with the alignments that passed filtering and exclude the problematic loci. Once the complete collection of filtered loci has been identified, final alignments are generated (6). If diplotype sequence data was used and heterozygous positions coded according to IUPAC format, concatenated (7) and SNP (8) alignments can be generated if required.

***Missing data – within alignments***

The effect of missing data on phylogenetic inference is not well understood and contradicting opinions coexist 9 ^,^ 10 ^,^ 11. Phylogenetic estimation is likely unbiased with large numbers of loci, if there are no systematic differences in sequence length between individuals for any given locus. However, the maximum-likelihood (ML) estimation might cluster individuals by sequence length rather than sequence similarity for complete positions, when missing data are non-randomly distributed and specific individuals have systematically shorter contigs or are completely missing for specific loci. At sites with missing data, the probability of observing an ‘A’, ‘T’, ‘C’ or ‘G’, is set to 1 and ML will group taxa together for which there is more signal and less uncertainty (Stamatakis, pers. comm.). Thus the effect of missing data is not limited to small sequence datasets but should also be accounted for and characterized in large-scale datasets. With the development of EAPhy, I do not advocate to discard or include incomplete sites but rather provide the opportunity to account for missing data by generating datasets where different filtering criteria have been enforced.

Missing data within individual sequences are particularly prevalent at the beginning and end of alignments (‘jagged edges’), since individual contig sequences often differ in length. Once alignments have been constructed using an existing aligner (e.g. Muscle 22), EAPhy will first address missing data by processing alignments in accordance with stringency criteria specified in the configuration script (Fig. 2). First, potential gaps within individual sequences are removed to yield long consecutive sequences (Fig. 2.1). EAPhy converts all sequence alignments into amino acid alignments and then uses a ‘jump-sliding window’ approach to assess the presence of potential non-consecutive sequence stretches that are often prevalent at the start/end of individual sequences. A jump-sliding window approach was developed since a conventional sliding window approach would remove the complete individual sequence if the first window would contain more missing data than allowed. Each window is assessed on the presence of amino acid sequence gaps and if a window contains more gaps than allowed, the complete window is removed for that individual sequence. In-frame gaps (i.e. triplet insertions) are retained if the amount of inserted codon gaps per window does not contain more missing codons than allowed. The window then ‘jumps’ a sequence distance of half the window size plus one codon and the process reiterates. By converting a nucleotide alignment in its amino acid equivalent, EAPhy specifically takes into account the coding-codon character of exonic sequences. When nucleotide sequence data is removed by codon, the remaining sequence is still in correct frame and codon position can still be inferred for each nucleotide position.

After individual sequences have been trimmed for missing data, EAPhy then assesses missing data between individuals by evaluating the amount of missing data for each amino acid alignment column (Fig. 2.2). The algorithm used is similar to the jump-sliding window approach, but now focuses on the amount of missing data within each amino acid alignment column. The window-length of amino acid columns and the amount of missing data allowed within each column, can be specified in the configuration script. The algorithm evaluates for each amino acid column whether the amount of individuals with missing data exceeds the cut-off specified. If more than half of the columns in a given window have more missing data than allowed, the columns in the first half of the window are removed from the alignment. The window then ‘jumps’ a sequence distance of half the window size plus one codon and the process reiterates. Amino acid columns at the end of alignments are removed, if they have not been evaluated but the specified window length exceeds the number of remaining columns. When alignments have been filtered for missing data within and between individuals, EAPhy evaluates the presence of single nucleotide insertions, by assessing the frequency of sequenced individuals for each nucleotide alignment column. If the number of sequenced individuals is below a user specified cut-off, the site is assumed to be a sequencing error and removed from the alignment.

**An exemplary overview of the three main filtering steps conducted during alignment filtering and quality assessment:**
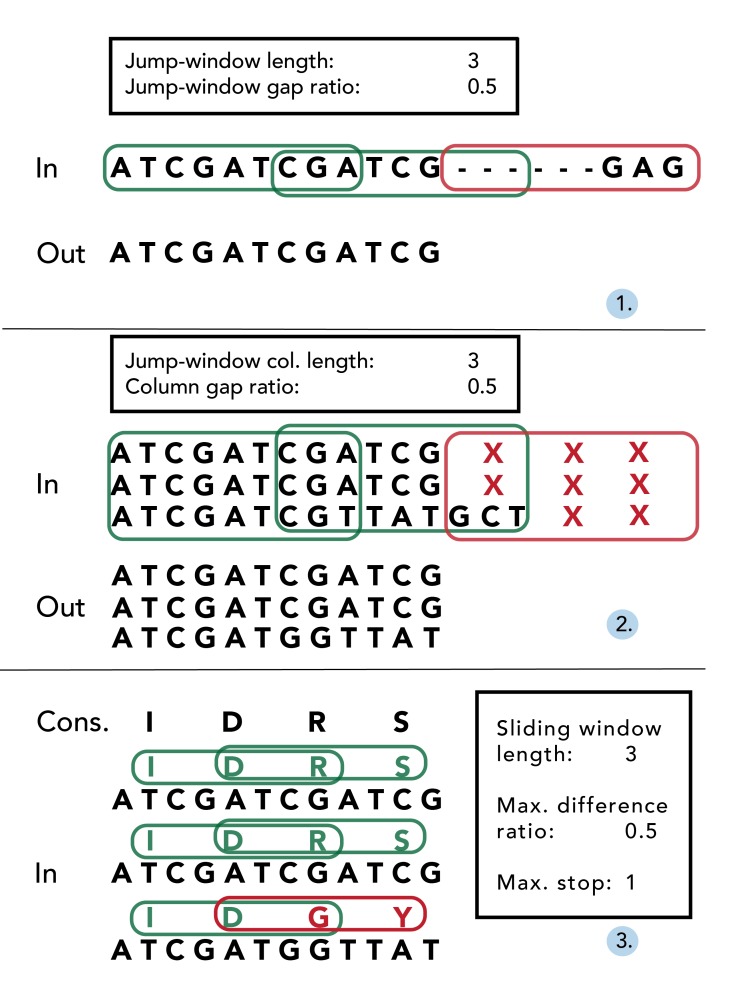
First, potential gaps within individual sequences are removed to yield long consecutive sequences (1). By converting nucleotide codons into amino-acids, the number of missing amino acids is assessed for each window using a ‘jump-sliding-window’ approach. If less amino-acids are missing for a given window than the specified ‘jump-window gap ratio’, the complete corresponding nucleotide stretch is retained (see green frames). If more amino acids are missing for a window, the complete nucleotide stretch is removed (see red frame). Secondly, the amount of missing data for each amino-acid alignment column in a given window is quantified (2). If more than half of the amino acid columns in a window miss more individuals than the specified ‘column gap ratio’, all corresponding nucleotide columns are removed (see red frame). Lastly, the quality of the resulting alignment is assessed, by comparing each individual sequence to a consensus sequence (3). Following a common sliding window approach for each individual sequence, the number of amino acids identical to the consensus is quantified. If, for each window, the number of amino acids distinct is less than the specified ‘difference ratio’, the alignment is retained (see green frames). If for any individual within an alignment, a window would fail this criterion, the alignment is flagged for visual inspection. In addition, for each sequence the number of stop-codons is quantified and if any individual sequence contains more stop-codons than a specified cut-off number (e.g. > 1), the alignment is also flagged for visual inspection.

***Alignment quality***

Once each alignment has been filtered for missing data, EAPhy then inspects the alignment quality by translating the nucleotide sequences and evaluating the resulting amino acid alignment (Fig. 2.3). First, if the number of stop codons for any individual sequence exceeds a user specified cut-off value (e.g. > 1), the alignment is flagged for visual inspection. Subsequently, a general consensus sequence is estimated for each alignment, and each individual sequence is compared to the consensus sequence in a ‘normal’ sliding-window approach. The window length is specified in the configuration script and each individual sequence is compared to the consensus sequence by sliding window. For each window, the number of amino acids distinct from the consensus is quantified and if greater than the proportion specified in the configuration script, the alignment is flagged for visual inspection.

Finally, phylogenetic inference is dependent on the comparison of orthologous genetic markers and comparing potential paralogous loci might yield confounded estimates of relationship. EAPhy assumes that the sequenced contigs for each locus are orthologous but has an additional option to potentially identify paralogous loci, by identifying markers with excessive levels of average individual heterozygosity. The user can inspect the distribution of average individual heterozygosity across all loci and based on this observation make an informed decision whether to exclude a certain percentage of loci with the highest level of average individual heterozygosity.

***Concatenation and SNP selection***

After visual inspection and filtering of flagged alignments, the collection of final high quality alignments can then be used for a variety of phylogenetic estimation methods. Gene trees can be inferred based on single alignments and a concatenated maximum likelihood tree can be estimated based on all alignments combined. Since all alignment filtering was conducted by codon, each nucleotide can still be assigned its correct codon position. PartitionFinder 23 ****estimates the most optimal partitioning scheme across all sequence positions and appropriate substitution model for each partition. A PartitionFinder input file is automatically created with each gene and codon position of the concatenated alignment specified.

In addition to sequence-based alignments, EAPhy will also generate concatenated alignments that include polymorphic sites exclusively. SNAPP 24 is a species tree method that uses unlinked biallelic markers, instead of sequence-based alignments, and EAPhy can generate alignments with a biallelic SNP randomly sampled from each locus. It will verify whether polymorphic sites are biallelic and neglect polymorphic sites with more than two allelic states. Alternatively SNP alignments can be constructed where every single SNP is considered, regardless of allele count, or with all SNP’s across all loci concatenated. If a study is geared towards recovering population structure, such alignments can be used in analyses that model allele frequencies (e.g. 25).

***Missing data – number of sequenced individuals***

Sequencing success can vary among individual samples. If specific individuals are systematically underrepresented and miss data for many loci, it is possible that the phylogenetic placement of such taxa is ambiguous and the investigator would prefer to exclude these samples. Thus, the potential impact of missing individuals across loci should be accounted for. EAPhy attempts to highlight where this is likely by: a) providing alternative datasets with different numbers of missing individuals allowed and b) providing summary statistic output files quantifying the number of loci sequenced for each individual. This enables the investigator to further explore the potential effects of missing data on phylogenetic inference.

## In Summary

The first objective of developing EAPhy was to provide a flexible and rigorous tool to generate reliable alignments, while minimizing the need for extensive visual inspection. Secondly, EAPhy was designed to allow filtering criteria for missing data to vary and investigate the impact of missing data on phylogenetic estimation. Lastly, EAPhy creates a large number of desired input formats for subsequent analysis, enabling the exploration of distinct inference methods. Negating the effort of manual alignment filtering and processing, EAPhy will hopefully stimulate further research into the potential consequences of applying alternative criteria for missing data and datatype, and how this might ultimately result in (in)congruent estimates of phylogenetic relationships across methods. The simultaneous development of novel molecular approaches to sequence orthologous genetic markers and bioinformatic methods to analyze such data, will ultimately provide us with the tools to generate a phylogenetic framework for all taxa across the tree of life.

## Competing Interests

The author has declared that no competing interests exist
